# How to reduce radiation exposure during hand surgery: use of a kidney dish to protect the surgeon’s hands

**DOI:** 10.1308/003588412X13171221591259i

**Published:** 2012-05

**Authors:** EAO Lindisfarne, A Baskaradas, K Kunasingam

**Affiliations:** Brighton And Sussex University Hospitals NHS TrustUK

## BACKGROUND

In-beam radiation exposure from the image intensifier to surgeons’ fingers during hand surgery has been measured as an average of 20mrem per case.^1^ For comparison, a chest x-ray results in approximately 20mrem exposure to the patient. Surgeons are advised to use techniques to minimise radiation exposure to their hands.^2^ We describe the novel use of kidney dishes for this purpose during hand surgery.

## TECHNIQUE

**Figure 1:**
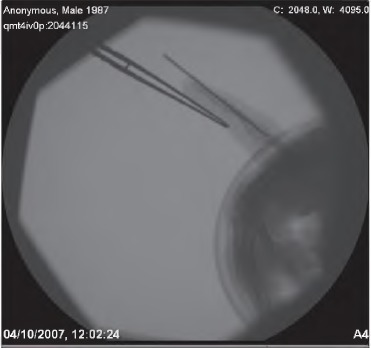
Lateral x-ray of finger with radiolucent kidney dish

**Figure 2:**
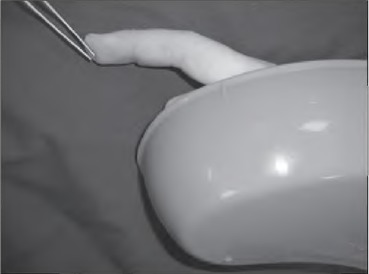
Kidney dish used to produce an image similar to the x-ray in Figure 1

A cheap and readily available plastic kidney dish is used to retract fingers (Fig 1). It is radiolucent and can be held directly over the imaging field without obscuring the images or affecting their quality. The blunt edges can be used on soft tissue without fear of damage. Figure 2 shows how a kidney dish has been used to produce a lateral image similar to the x-ray in Figure 1.

## DISCUSSION

Image intensifies are used to aid correct placement of K-wires and plates during hand surgery for trauma. Lead aprons reduce radiation to the operator but leave the surgeon’s hands exposed. Furthermore, the surgeon’s own hands are often used to isolate the patient’s finger for imaging and therefore lie in close proximity to the imaging field. Surgeons often witness their own fingers being x-rayed. Use of the plastic kidney dishes allows good control of the patient’s fingers and reduces operator exposure to radiation.

## References

[1] Singer G, Radiation exposure to the hands from mini C-arm fluoroscopy J Hand Surg Am 2005 30 795 797 16039374 10.1016/j.jhsa.2005.01.007

[2] Athwal GS, Bueno RA, Wolfe SW., Radiation exposure in hand surgery: mini versus standard C-arm J Hand Surg Am 2005 30 1,310 1,316 16344194 10.1016/j.jhsa.2005.06.023

